# Supplementary material for: Promoting favorable interfacial properties in lithium-based batteries using chlorine-rich sulfide inorganic solid-state electrolytes
Source: Nat Commun. 2022 Apr 7;13:1909. doi: 10.1038/s41467-022-29596-8 (PMC8989881; doi:10.1038/s41467-022-29596-8)
Supplement: Supplementary file 1 — Supplementary Information [file 41467_2022_29596_MOESM1_ESM.pdf]

Supplementary Information

Promoting favourable interfacial properties in lithium-based batteries using chlorine-rich sulfide inorganic solid-state electrolytes

Dewu Zeng,[1] Jingming Yao,[1] Long Zhang,[1*] Ruonan Xu,[1] Shaojie Wang,[2] Xinlin Yan,[3] Chuang Yu,[4] Lin Wang[2]

[1]Clean Nano Energy Center, State Key Laboratory of Metastable Materials Science and Technology, Yanshan University, Qinhuangdao, Hebei 066004, China
[2]Center for High Pressure Science (CHiPS), State Key Laboratory of Metastable Materials Science and Technology, Yanshan University, Qinhuangdao, Hebei 066004, China
[3]Institute of Solid State Physics, Vienna University of Technology, Wiedner Hauptstr. 8-10, 1040 Vienna, Austria
[4]State Key Laboratory of Advanced Electromagnetic Engineering and Technology, School of Electrical and Electronic Engineering, Huazhong University of Science and Technology, Wuhan 430000, Hubei, China

*Corresponding author E-mail: lzhang@ysu.edu.cn

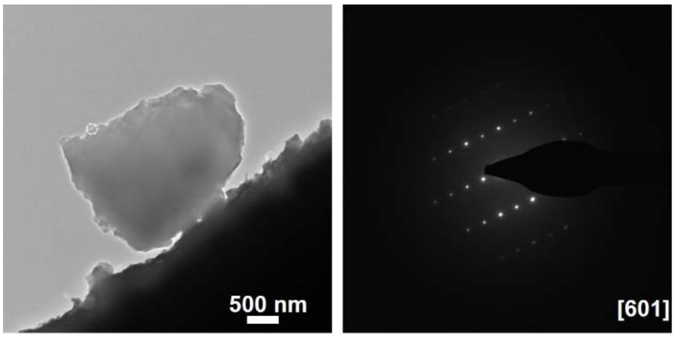

Supplementary Figure 1. TEM image of CI-10 and the corresponding SAED pattern.

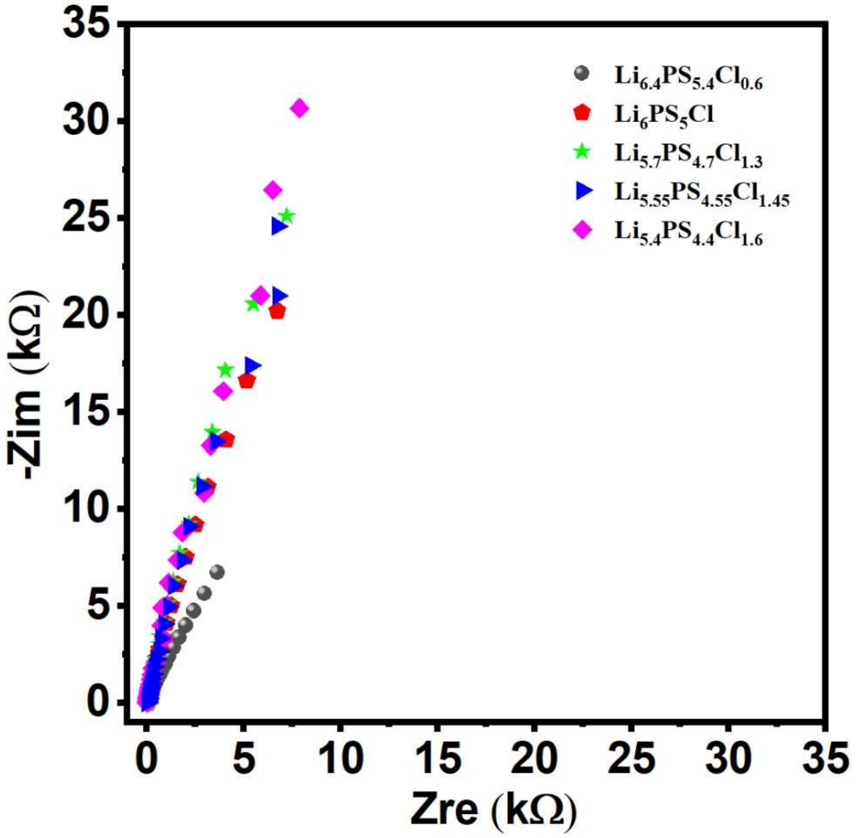

Supplementary Figure 2. The full impedance plots of $Li_{7-x}PS_{6-x}Cl_x$ measured on In|SE|In cells at 24 °C.

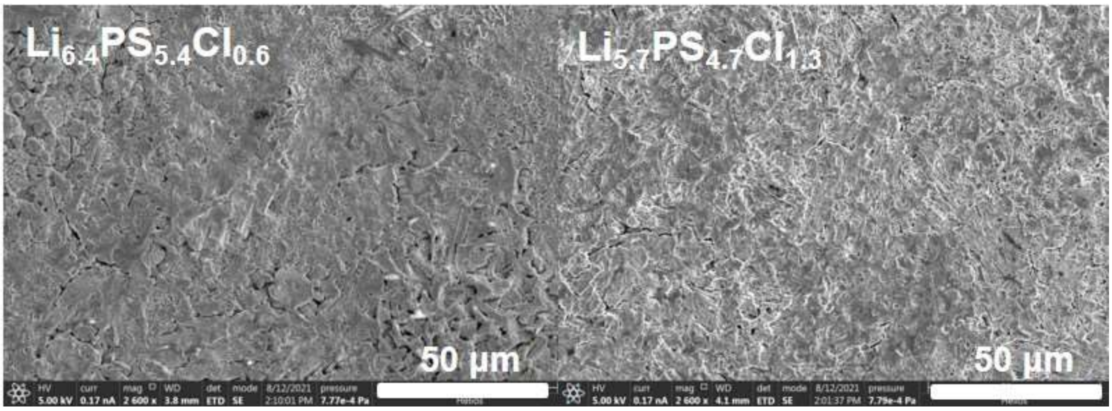

Supplementary Figure 3. SEM surface images of the pristine Cl-06 and Cl-13 argyrodites cold-pressed from powders. The two pellets show comparable morphology.

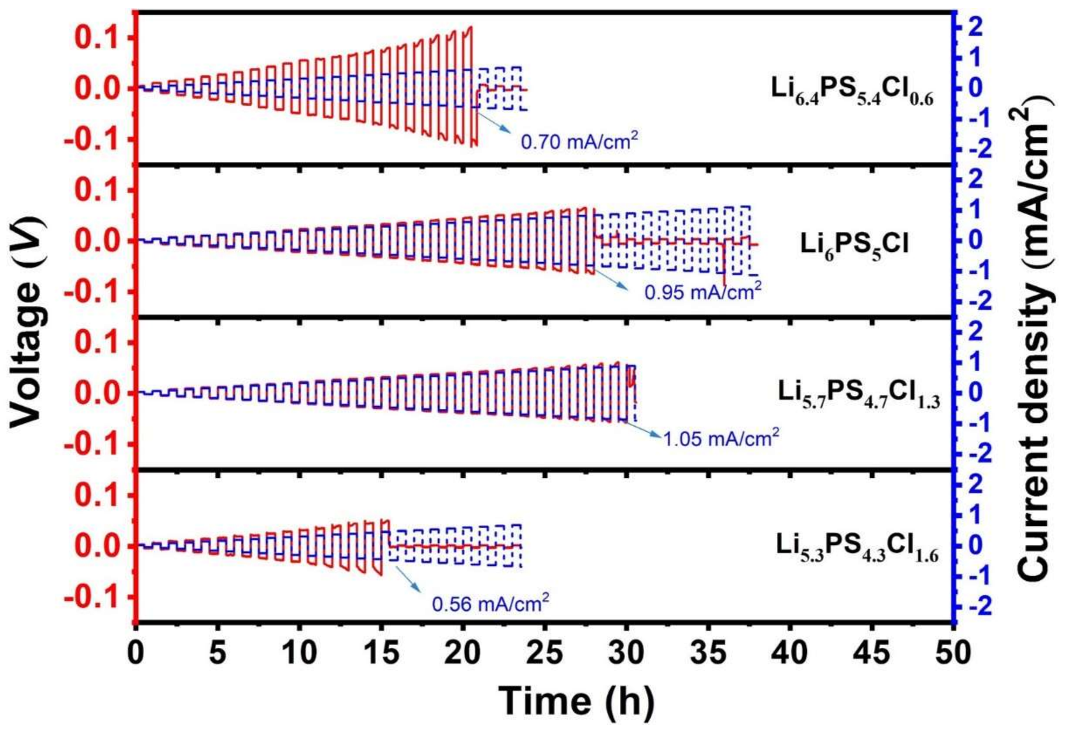

Supplementary Figure 4. CCD curves for $Li_{7-x}PS_{6-x}Cl_x$ (x = 0.6, 1.0, 1.3, 1.6) with different chlorinities tesed at 24 ± 4 °C. The arrows indicate the short circuit points. A moderate chlorinity with x = 1.3 shows the highest CCD, while the highest chlorinity with x = 1.6 shows the lowest CCD.

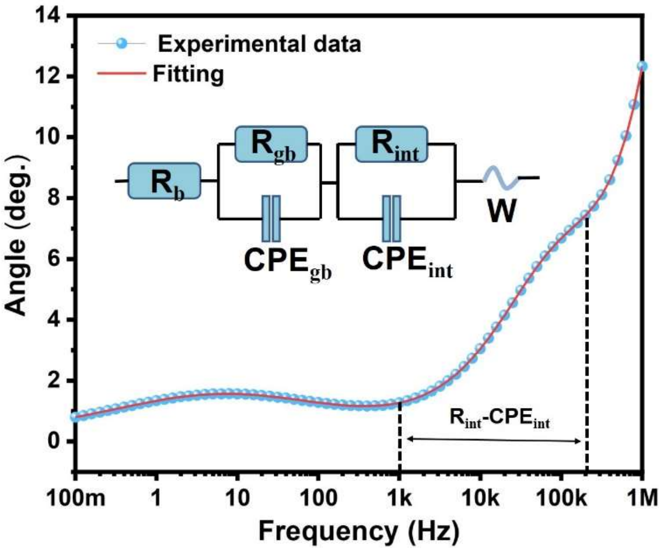

Supplementary Figure 5. Representation of the impedance signal in a phase angle

Bode plot of Li|Cl-13|Li cell afte the 200th cycle (Figure 4c).

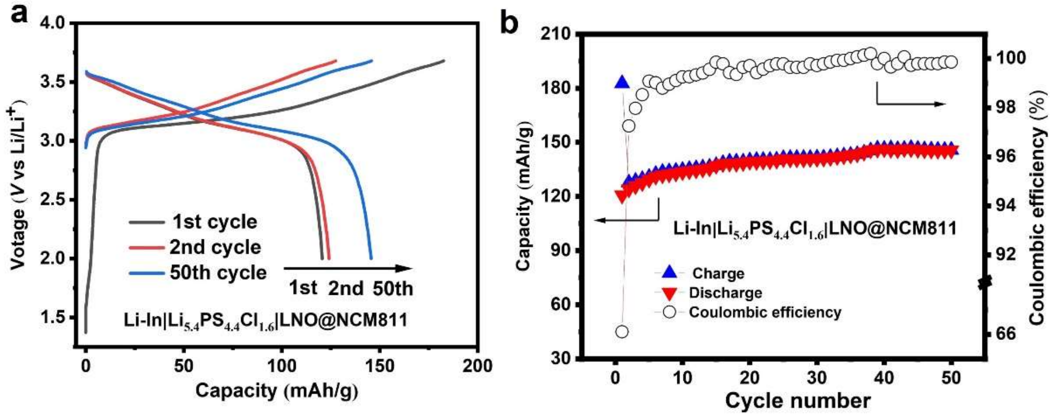

Supplementary Figure 6. Charge/discharge profiles (**a**) and capacity and Coulombic efficiency (**b**) of the Li-In|Cl-16|LNO@NCM811 (Li: 5 wt%) cell run at 0.5 mA/cm^2 at 24 ± 4 °C.

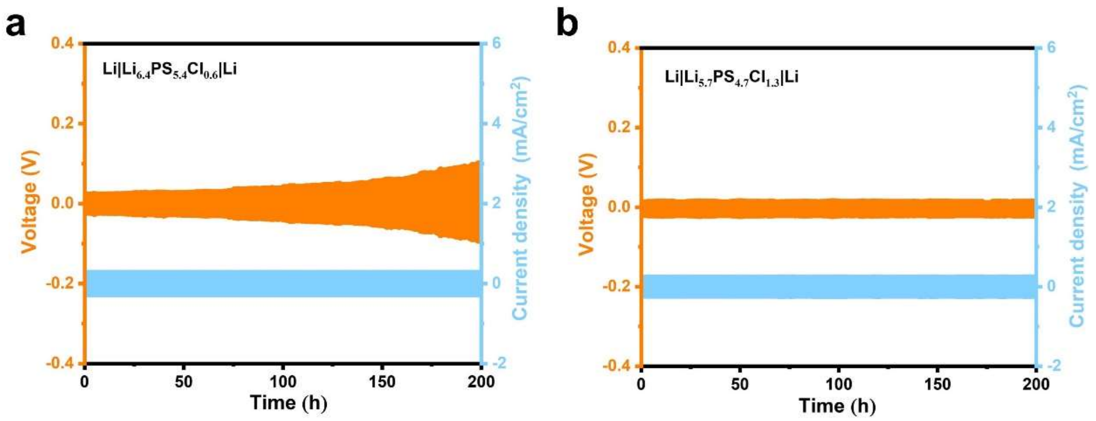

Supplementary Figure 7. Li plating/stripping cycled at 0.25 mA/cm^2 at 24 ± 4 °C for 200 cycles on Li||Li symmetric cells using Cl-06 (a) and Cl-13 (b) SEs. After cycling, the cells were detached for further investigations on the Li|SE interface.

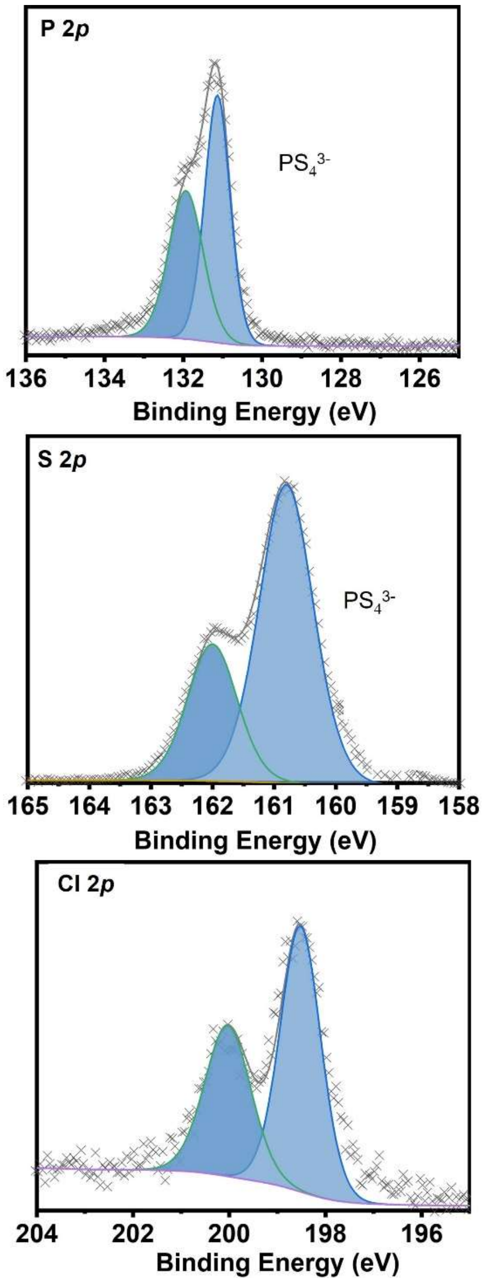

Supplementary Figure 8. XPS spectra of the as-prepared Cl-06.

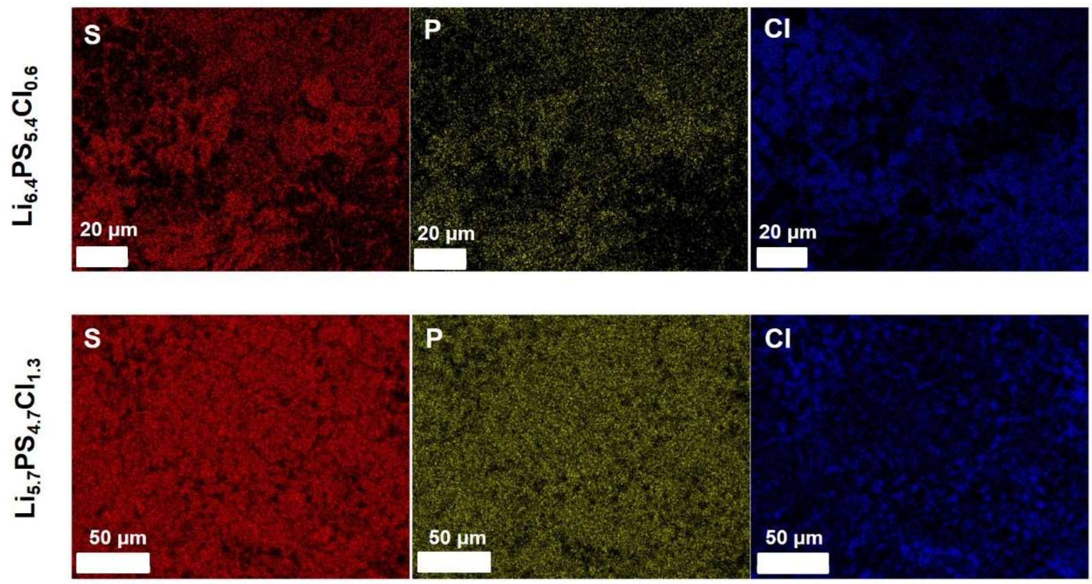

Supplementary Figure 9. EDS mapping images of S, P, Cl elements on the fracture surface of Li|Cl-06 (**a**) and Li|Cl-13 (**b**) after Li plating/stripping cycling. The SEM images of the detection regions are shown in Figure 8a. The detections were performed on the Li|SE interface for Li||Li symmetric cells after plating/stripping cycling using Cl-06 (200 cycles, Supplementary Figure 7) and Cl-13 (400 cycles, Figure 3d) SEs. Agglomeration of the decomposition products are observed from the former but not from the latter.

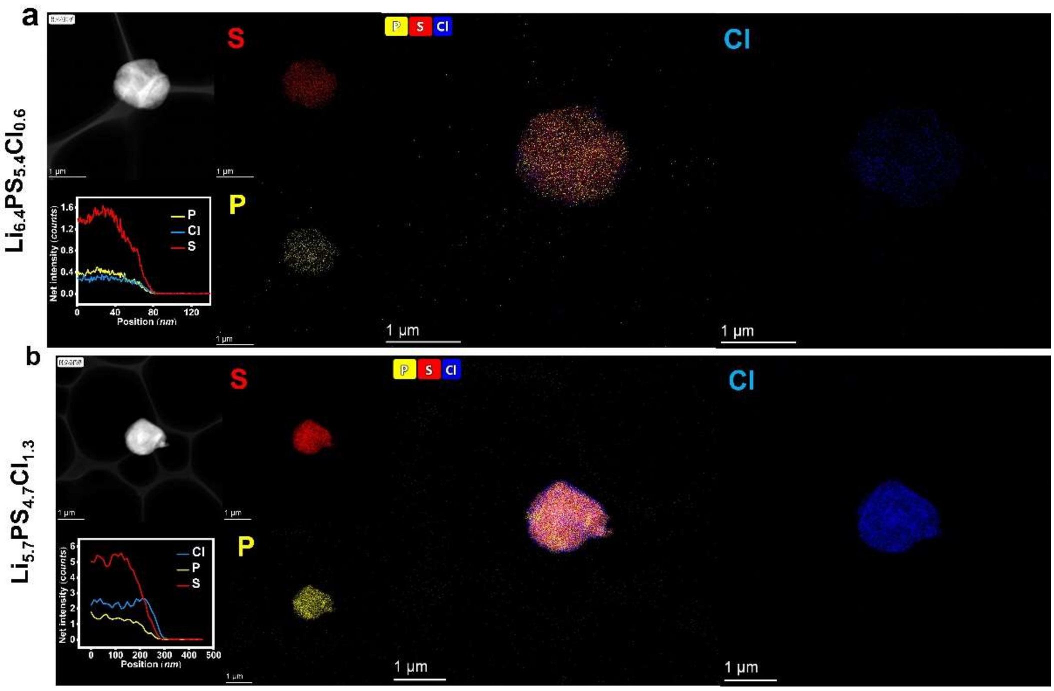

Supplementary Figure 10. STEM-HAADF and EDS mapping images of Cl-06 (**a**) and Cl-13 (**b**). A homogeneously elemental distribution is seen from Cl-06, while a LiCl nanoshell with a thickness about 20 nm is found for Cl-13.

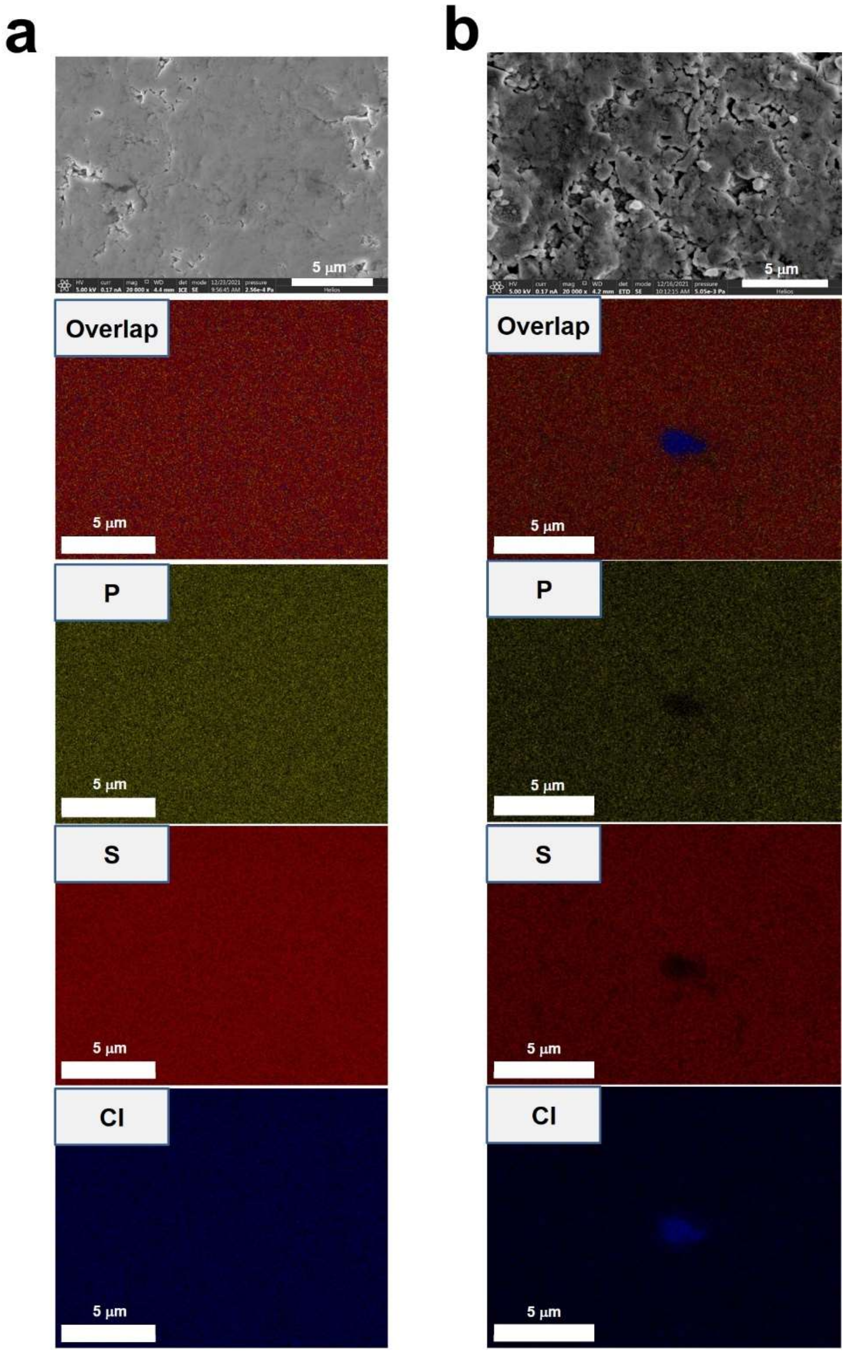

Supplementary Figure 11. SEM-EDS images of the pellet cold-pressed from the as-synthesized Cl-13 powders. The pellet was tested more than 7 regions (20,000×). **a**, Selected images on the region with homogeneous elemental distribution. **b**, The only region with an agglomerated LiCl particle.

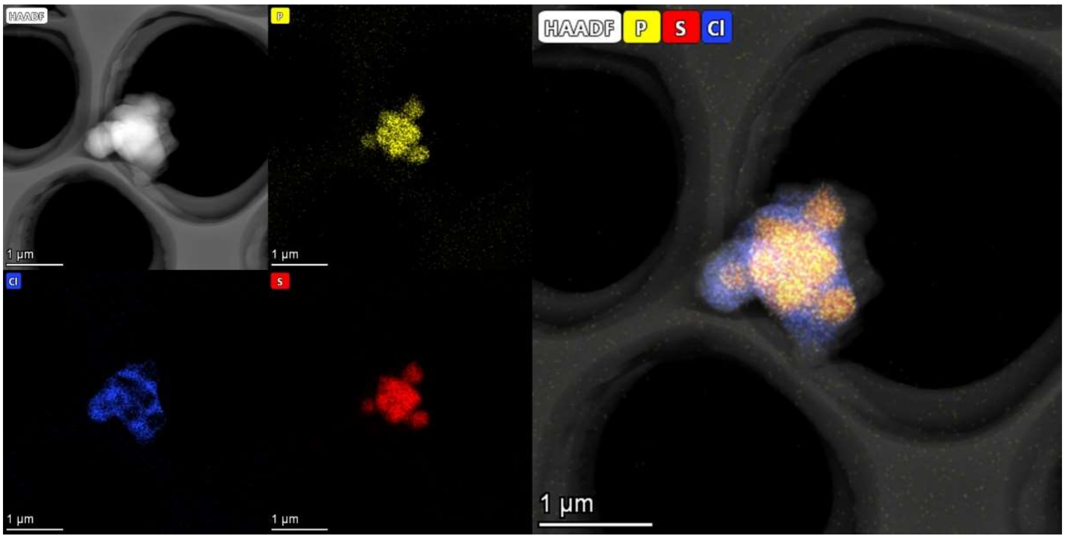

Supplementary Figure 12. STEM-HAADF and EDS mapping images for a repeatable detection on another Cl-16 powder.

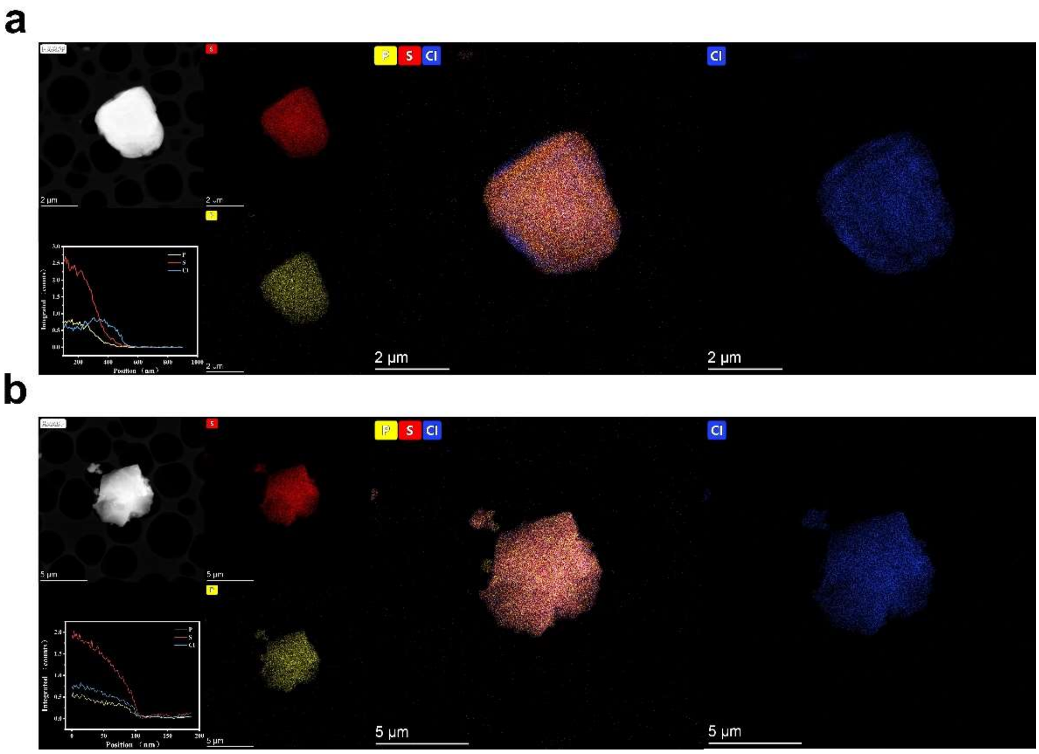

Supplementary Figure 13. STEM-HAADF and EDS images of Cl-16 prepared with different cooling processes by (**a**) cooling the sample to 400 °C and then quenching in the ice water, and (**b**) directly quenching in the ice water.

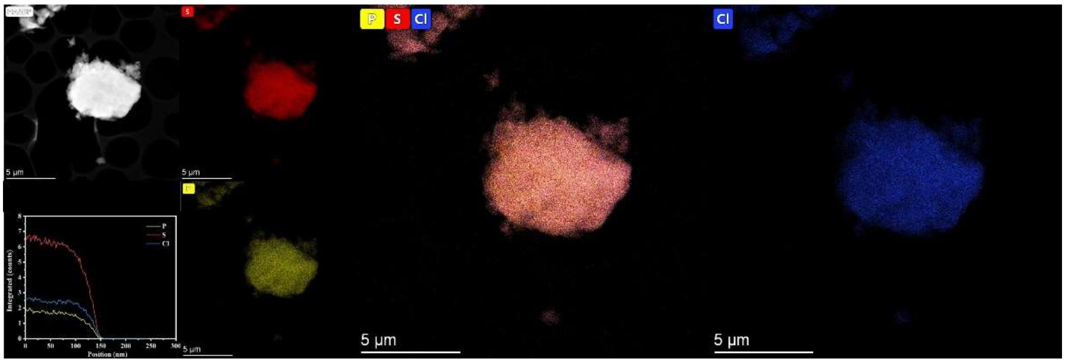

Supplementary Figure 14. STEM-EDS of Cl-16 after Li plating/stripping for 400 cycles (Figure 3d in the main text).

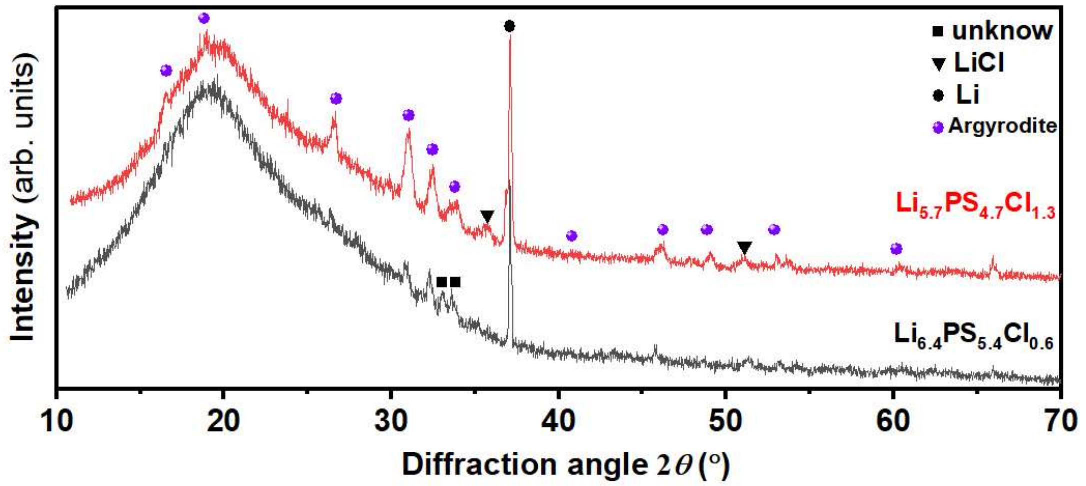

Supplementary Figure 15. The XRD profiles detected from the Li|SE interface on Li||Li symmetric cells using Cl-06 and Cl-13 SEs after Li plating/stripping for 200 cycles (Supplementary Figure 7).

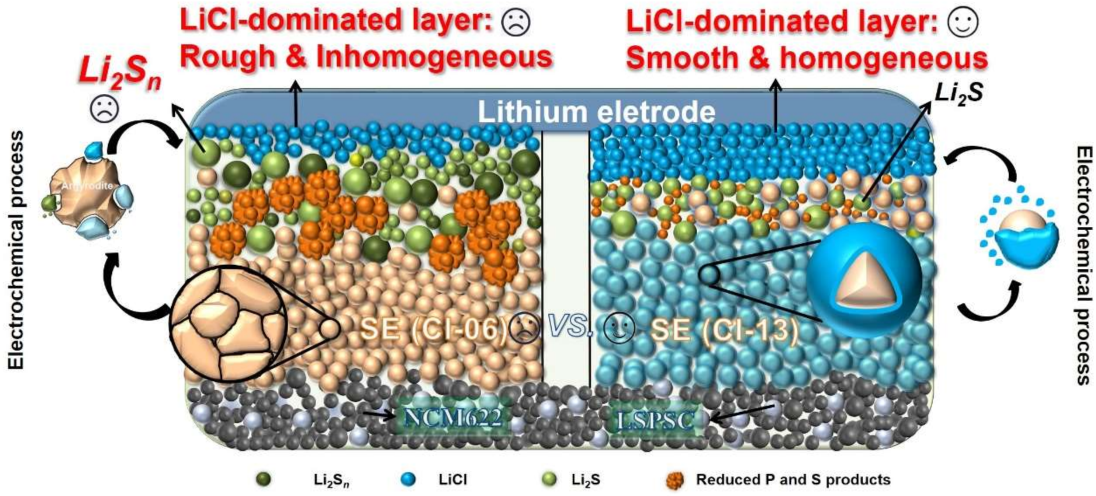

Supplementary Figure 16. Schematic depicting the advantages of the Cl-rich (Cl-13) compared with the Cl-poor (Cl-06) argyrodites and the formation of the differentiated interface of Li|SE.

Supplementary Table 1. The ionic conductivity, electronic conductivity, lattice parameters, and LiCl volume fraction of the as-prepared Li argyrodites with various chlorinities.

**Nominal composition**	$Li_{6.4}P4_{5.4}Cl_{0.6}$	Li_6PS_5Cl	$Li_{5.7}PS_{4.7}Cl_{1.3}$	$Li_{5.55}PS_{4.55}Cl_{1.45}$	$Li_{5.4}PS_{4.4}Cl_{1.6}$
**Ionic conductivity (mS/cm)**	1.6	3.6	5.3	6.1	8.8
**Electronic conductivity (S/cm)**	4.2×10^{-11}	3.75×10^{-11}	1.04×10^{-11}	-	1.3×10^{-12}
**Lattice parameters (Å)**	9.890(1)	9.841(1)	9.817(1)	9.805(1)	9.788(1)
**LiCl fraction (vol%)**	0	0	2.7(2)	3.2(5)	4.4(5)

Supplementary Table 2. R_{int} values and corresponding errors acquired by fitting the impedance spectra (Figure 2a-d) with the equivalent circuit displayed in Figure 2e. The time-resolved impedance spectra were collected for every 100 h.

Nominal composition \ Cycle number		pristine	100h	200h	300h	400h	500h	600h
**$Li_{6.4}PS_{5.4}Cl_{0.6}$**	**R_{int} (Ω)**	209	591	2175	3671	5129	6247	9093
	**Error (%)**	7.9	8	2.7	1.9	5.71	9.6	4.4
**Li_6PS_5Cl**	**R_{int} (Ω)**	61	144	180	265	320	422	568
	**Error (%)**	3.3	6.6	5.7	6.4	5.8	5.1	7.2
**$Li_{5.7}PS_{4.7}Cl_{1.3}$**	**R_{int} (Ω)**	50	81	127	182	284	346	486
	**Error (%)**	8.2	1.6	4.1	8.61	8.1	5.9	8.6
**$Li_{5.4}PS_{4.4}Cl_{1.6}$**	**R_{int} (Ω)**	19	82	95	102	135	175	256
	**Error (%)**	13.1	8.32	10.3	6.3	5.6	5.4	7.8

Supplementary Table 3. R_{int} values and corresponding errors acquired by fitting the impedance spectra with the equivalent circuit displayed in Supplementary Figure 6. The impedance spectra (Figure 4) were in situ measured from the Li plating/stripping cycling on Li|SE|Li symmetric cells for every 100 cycles.

Nominal composition \ Cycle number		0 (pristine)	100th	200th	300th	400th
$Li_{6.4}PS_{5.4}Cl_{0.6}$	R_{int} (Ω)	258	506	623	-	-
	Error (%)	0.001	0.001	7.793	-	-
Li_6PS_5Cl	R_{int} (Ω)	63	137	246	403	465
	Error (%)	0.002	5.760	0.003	9.340	10.490
$Li_{5.7}PS_{4.7}Cl_{1.3}$	R_{int} (Ω)	40	50	72	92	178
	Error (%)	0.009	2.167	0.238	12.131	9.061
$Li_{5.4}PS_{4.4}Cl_{1.6}$	R_{int} (Ω)	17	28	40	32	49
	Error (%)	15.993	0.007	0.009	24.160	28.040
